# Direct visualization of the charge transfer state dynamics in dilute-donor organic photovoltaic blends

**DOI:** 10.1038/s41467-024-53694-4

**Published:** 2024-11-14

**Authors:** Gareth John Moore, Florian Günther, Kaila M. Yallum, Martina Causa’, Anna Jungbluth, Julien Réhault, Moritz Riede, Frank Ortmann, Natalie Banerji

**Affiliations:** 1 Department of Chemistry, Biochemistry and Pharmaceutical Sciences, University of Bern, Bern, Switzerland; 2 Instituto de Física de São Carlos (IFSC), Universidade de São Paulo (USP), São Carlos, Brazil; 3 Instituto de Geociências e Ciências Exatas (IGCE), São Paulo State University (UNESP), Rio Claro, Brazil; 4 Center for Advancing Electronics Dresden, Technische Universität, Dresden, Germany; 5 Clarendon Laboratory, Department of Physics, University of Oxford, Oxford, UK; 6 Department of Chemistry, TUM School of Natural Sciences, Technische Universität München, Garching b, München Germany

**Keywords:** Solar cells, Electron transfer

## Abstract

The interconversion dynamics between charge transfer state charges (CTCs) and separated charges (SCs) is still an unresolved issue in the field of organic photovoltaics. Here, a transient absorption spectroscopy (TAS) study of a thermally evaporated small-molecule:fullerene system (α6T:C_60_) in different morphologies (dilute intermixed and phase separated) is presented. Spectral decomposition reveals two charge species with distinct absorption characteristics and different dynamics. Using time-dependent density functional theory, these species are identified as CTCs and SCs, where the spectral differences arise from broken symmetry in the charge transfer state that turns forbidden transitions into allowed ones. Based on this assignment, a kinetic model is formulated allowing the characterization of the charge generation, separation, and recombination mechanisms. We find that SCs are either formed directly from excitons within a few picoseconds or more slowly (~30–80 ps) from reversible splitting of CTCs. These findings constitute the first unambiguous observation of spectrally resolved CTCs and SCs.

## Abstract (web summary)

The interconversion dynamics between charge transfer state charges and separated charges remains an unresolved issue. Here, the authors spectrally resolve those charges and report a kinetic model to reveal the charge generation, separation, and recombination mechanism in α6T:C_60_ systems.

## Introduction

Power conversion efficiencies (PCEs) of organic photovoltaics (OPVs) continued to rise over the last few years with the introduction of novel materials and blend morphologies^1–5^. These developments were enabled by the continuous improvement in understanding the photon-to-current conversion. Due to complex energetics, interfaces and morphologies, however, there is still much to be learned, particularly about the charge generation process. Ultrafast techniques, such as transient absorption spectroscopy (TAS), bring invaluable insights^6–9^. Unfortunately, TAS is typically unable to spectrally distinguish interfacial charge transfer state charges (CTCs) from separated charges (SCs). A more indirect way to determine the dynamics of CTCs and SCs from TAS data is to model their fluence-dependent dynamics^10,11^, but the underlying assumption that CTCs do not convert to SCs has been questioned by subsequent work^12,13^. Therefore, it remains challenging to directly follow charge transfer (CT) state dynamics and charge separation, so that these processes are still highly debated in the literature^12,14–21^.

**Fig. 1:**
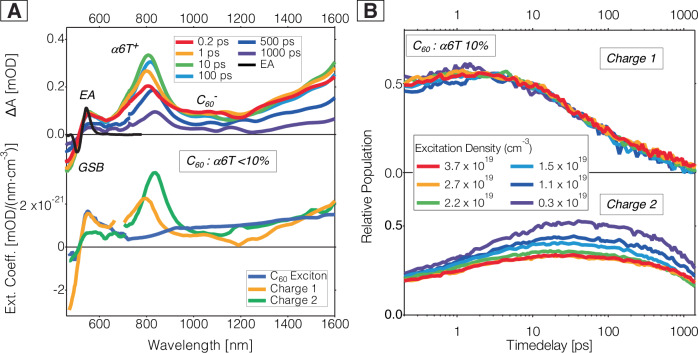
Transient absorption (TA) data of ‘dilute-donor’ *α*6T:C_60_ blends. **A** Top panel: TA spectra of a ‘dilute-donor’ α6T:C_60_ blend with a weight ratio of ~7% at selected time delays (0.2 ps, 1 ps, 10 ps, 100 ps, 500 ps, and 1 ns), with an excitation density of 3.3 × 10^18^ cm^−3^ at 610 nm. The visible part of the spectra (VIS, 470–720 nm) was measured on a different spectrometer than the near-infrared part (NIR2, 690–1600 nm) and was scaled to obtain continuous spectra. The oscillatory electro-absorption (EA) feature, measured at 6 V reverse bias on a neat C_60_ device, is illustrated as a black line overlaid with the spectra. Bottom panel: Spectral components of the MCR-ALS decomposition of the ~7% ‘dilute-donor’ α6T:C_60_ system, with the C_60_ Frenkel-type exciton (blue, held constant as in neat C_60_ film), the first charge component (orange, Charge 1) and the second charge component (green, Charge 2), all expressed as the extinction coefficient (transient absorption signal divided by the species density and film thickness, see methods). **B** MCR-ALS dynamics of the Charge 1 component (top panel) and Charge 2 component (bottom panel) for a 10% α6T:C_60_ blend, divided by the different excitation densities shown in the legend (in cm^−3^). The corresponding TA spectra and MCR components are shown in Fig. S4.

Here, we investigate thermally evaporated thin films of electron-donating α-sexithiophene (α6T) with electron-accepting C_60_, intended for use as active layers in organic solar cells, with morphologies controlled by the donor:acceptor ratio or by intentionally separating the materials in a bilayer (Fig. 1A). The reliability of evaporated small molecule solar cells makes them commercially successful alternatives to solution-processed polymer-based systems^22,23^. We consider two scenarios. First, bulk heterojunctions (BHJs) with more than 90% of the fullerene acceptor blended with the small molecule donor, called ‘dilute-donor’ systems, create an intermixed morphology where donor molecules (or small clusters) are surrounded by acceptor molecules. These blends have been extensively characterized structurally in previous works, including spectroscopic techniques^24^, and microstructural probes via X-ray reflectivity (XRR) and grazing incidence X-Ray scattering (GIWAXS)^25^. The homogeneous dispersion of the donor molecules at random orientations in the C_60_ matrix is further confirmed by the constant CT state energy at low α6T concentration^26^, and by the linear increase of CTCs with α6T content^24^, allowing us to disregard aggregation and to correlate charge dynamics to the amount of donor present in the films. Second, we contrast the intermixed case in the ‘dilute-donor’ blends to the completely phase-separated situation, achieved in the bilayer sample.

The analysis of the TAS data is accompanied by time-dependent density functional theory (TD-DFT) calculations. According to the obtained results, the absorption spectrum of the donor cation in α6T:C_60_ systems changes in the proximity of the acceptor anion (i.e., in the CT state), which is caused by symmetry breaking. This renders CTCs and SCs spectrally distinguishable by TAS. Kinetic modelling of the corresponding TAS components provides a direct quantification of the CT state dynamics, including the hole transfer (HT) process, the CT state lifetime, the separation into SCs, and sub-nanosecond recombination processes at varied excitation densities. It is found that excitons dissociate directly into CTCs and SCs, in equal amounts. The CTCs then undergo a reversible conversion to SCs. The back-conversion from SCs to CTCs is involved in recombination, that can occur in less than a nanosecond in the ‘dilute-donor’ systems due to trapping by the immobile hole, while bimolecular recombination of SCs occurs at high fluencies.

## Results

### Time-resolved spectroscopy of α6T:C_60_ thin films

The preparation of the thin films (50 nm thickness) by thermal evaporation is described in detail in the methods section below. The dilution of the donor (<10%) was chosen because of the homogeneous dispersion of α6T within large C_60_ clusters^8,24,27^. The two ‘dilute-donor’ α6T:C_60_ systems with 5% and 10% donor content by weight show a similar open-circuit voltage (Voc = 0.93 and 0.92 V, respectively), short-circuit current density (Jsc = 6.07 and 6.14 mA/cm^2^) and power conversion efficiency (PCE = 3.18% for both blends)^24,28^, which are all significantly larger than for the phase-separated bilayer (PCE 0.06–0.15%)^29^. Transient absorption (TA) spectra of the α6T:C_60_ systems were measured with excitation at 610 nm. The α6T donor does not absorb at this wavelength, such that the C_60_ acceptor is selectively excited (Fig. S1). Moreover, we have previously demonstrated that pumping C_60_ at 610 nm results in Frenkel-type excitons (TA spectra of neat C_60_ film are shown in Fig. S2 for reference)^30,31^.

In the top panel of Fig. 1A, the TA spectra of a ‘dilute-donor’ α6T:C_60_ blend (containing ~7% donor), are shown. The data for 5% and 10% dilutions is depicted in Figs. S3–S4 and show similar features. Three different spectrometers were used to cover different spectral regions and show consistent results (VIS: 470–720 nm, NIR1: 840–1150 nm, NIR2: 690–1600 nm). In the TA spectra, we see a mixture of signatures of both C_60_ Frenkel-type excitons and charge species. In agreement with previous reports, the excitons show a small negative ground state bleaching (GSB) at wavelengths lower than 500 nm as well as excited state absorption (ESA) peaks at ∼550 nm and ∼950 nm, and a broad band extending throughout the near infra-red (NIR) range^6,30^. The charge features consist of the GSB, the C_60_ anion with a broad NIR signature around 1000 nm^30,32,33^, the α6T cation absorption at 650–900 nm^8,34^, and a further band (likely delocalized charges) rising towards 1600 nm. The GSB and the ESA peaks are superimposed with an additional oscillatory signature assigned to electro-absorption (EA) (negative lobe at 495 nm and positive lobe at 555 nm, black line in Fig. 1B bottom left). EA is the result of the electric field between the electron and hole that causes a Stark shift in neighbouring C_60_ clusters^6,30^. Evidence of a long-lived charge signal is seen at times as long as 1 ns.

Importantly, a strong and gradual shift over time of the α6T cation band (from ∼820 to 840 nm) and a slight narrowing are observed in the TA spectra. The lower panel of Fig. 1A shows the spectral components resulting from Multivariate Curve Resolution with Alternating Least Squares (MCR-ALS) decomposition of the above TAS data (see Figs. S3–S5 for the other samples)^35,36^. The TA spectra were best decomposed into three components (confirmed by Singular Value Decomposition, Figure S6) representing the C_60_ Frenkel exciton, a first charge species (Charge 1, C1) and a second charge species (Charge 2, C2). The main difference between the C1 and C2 components is the significant red shift of the α6T cation band of the C2 component, which matches with the gradual shift seen in the TA spectra. We conclude that the perceived red shift of the α6T cation band is a result of the two charge species evolving with different dynamics (*vide infra*). Apart from the shift of the α6T cation band, other differences between the C1 and C2 spectra include a stronger EA signature for C1 that almost vanishes in C2, and a higher/broader C_60_ anion absorption for C2, suggesting more anion delocalization into C_60_ clusters^30^.

Figure 1B shows the dynamics of the C1 (top) and C2 (bottom) components from the MCR-ALS decomposition of a 10% α6T:C_60_ blend at different excitation densities. We see that the C2 charges are formed slower than the C1 charges. Moreover, the decay of the C1 dynamics shows almost no dependence on the excitation density within experimental error, pointing to monomolecular recombination of bound electron-hole pairs. On the contrary, the fluence-dependence for C2 indicates fast bimolecular recombination of separate charges, competing with the build-up of their population and leading to overall lower relative density (i.e., yield) at high fluencies.

**Fig. 2:**
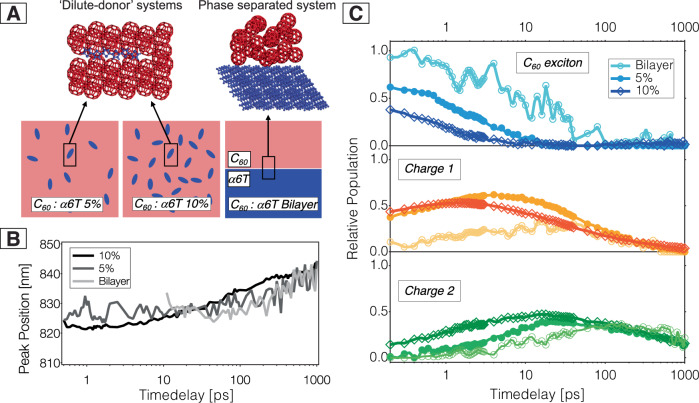
Temporal evolution of the excitons and charges in ‘dilute-donor’ and bilayer films. **A** Illustration of ‘dilute-donor’ (left) and phase-separated (right) systems, with the interface at a molecular scale shown on top and the longer-scale morphology below. **B** Temporal shift of the maximum of the α6T cation peak for the 5% and 10% blends and bilayer, obtained by Gaussian fitting of the TA band in the NIR 2 data. As charges are formed later in the bilayer, the analysis starts only at 10 ps. **C** Dynamics of the three components of the MCR-ALS decomposition in each of the blends and bilayer, with the exciton dynamics (top), the dynamics of Charge 1 (middle), and the dynamics of Charge 2 (bottom). Taken from the VIS/NIR1 data and divided by the excitation density of 1.1 ± 0.1 × 10^19^ cm^−3^ to give the relative population.

To investigate how the morphology of the α6T:C_60_ systems affects the evolution of the TA spectra, we have compared two ‘dilute-donor’ blends with different α6T content (5% and 10% by weight) to the completely phase-separated bilayer. The morphologies are shown schematically in Fig. 2A. The bilayer was fabricated in glass/α6T/C_60_ architecture and was excited from the C_60_ side (25 nm thickness of each the α6T and C_60_ layer). The corresponding TA spectra contain exciton and charge signatures similar as for the dilute blends (Fig. S5), but the C_60_ excitons dominate. Nonetheless, some charges are formed that persist as an offset, and the shift in the α6T cation band is clearly seen for the bilayer as well. To quantify the spectral red shift of the α6T cation band related to the two charge components, we fit a Gaussian to the band and plot the temporal evolution of the peak position in Fig. 2B for the three investigated samples. A pronounced and similar shift of about 20 nm is seen in both ‘dilute-donor’ blends as well as the bilayer. Moreover, a spectral narrowing occurs, as illustrated for the 10% blend in Fig. S7. We conclude that the presence of two distinct charge components is characteristic for the α6T:C_60_ system, independently of whether the donor and acceptor are intermixed or phase-separated.

Indeed, MCR-ALS analysis yields comparable spectral signatures for the C1 and C2 components of the dilute blends and bilayer (Figs. S3–S5). The relative dynamics of the spectral components, Frenkel excitons and charges (C1 and C2), are shown in Fig. 2C for an excitation density of 1.1 × 10^19 ^cm^−3^ at 610 nm. They are scaled by the extinction coefficient of the species and normalized by the excitation density. The C_60_ Frenkel-type excitons in the 5% and 10% blends completely disappear within a few picoseconds. However, excitons in the 5% blend have almost twice the initial population (62% compared to 38% for the 10% blend, at 0.2 ps) and survive longer. For both dilute blends, the excitons in C_60_ diffuse to the α6T molecules and undergo hole transfer (HT). As there is more α6T in the 10% blend than the 5% blend (Fig. 2A), the average path needed to find an α6T site is shorter resulting in faster quenching of the excitons^24^. Both C1 and C2 species are thus formed faster in the 10% blend than in the 5% blend. However, the rise of the C2 species is systematically delayed as compared to C1 for both dilute blends and reaches a maximum at around 20 ps when C1 already decays. Moreover, the C2 decay is slower than for the C1 species.

For the bilayer, the exciton signature lasts much longer, comparably as long as in a neat C_60_ film (150 ps lifetime)^30^. This indicates that the dynamics are dominated by excitons formed in the bulk of the C_60_ clusters that are not entirely quenched by the donor due to the complete phase separation between C_60_ and α6T^24^. Less than half of the exciton population undergoes slow HT after diffusion to the interface, leading to the gradual rise of the C1 and later the C2 component. The yield of C2 at long times is nevertheless similar as in the dilute blends and this component shows almost no recombination on the investigated time window of 1 ns.

### Spectral simulation of the α6T cation spectra

To study the origin of the two distinct charge species (C1 and C2) obtained from the decomposed TA spectra, we performed TD-DFT calculations to simulate the absorption spectra of charged thiophenes under different conditions (see methods section). In principle, the different spectral signatures could originate from two scenarios, namely (i) from CT states with different relative molecular orientations (face-on, tip-on, edge-on) between the α6T cation and C_60_ anion, or (ii) from whether the C_60_ anion is in the proximity of the probed α6T cation or not; which represents CTCs and SCs, respectively. The different scenarios considered in the TD-DFT simulations are therefore α6T cations in CT states, modelled by representing the C_60_ anion as a negative point charge at different positions with respect to the cation, and isolated α6T cations representative of SCs. Additional tests (beyond the point charge model for CTCs) explicitly include the C_60_ anion in constrained DFT (cDFT) and TD-DFT simulations by simulating the charge transfer state absorption, and corroborate the findings presented here (Fig. S8).

**Fig. 3:**
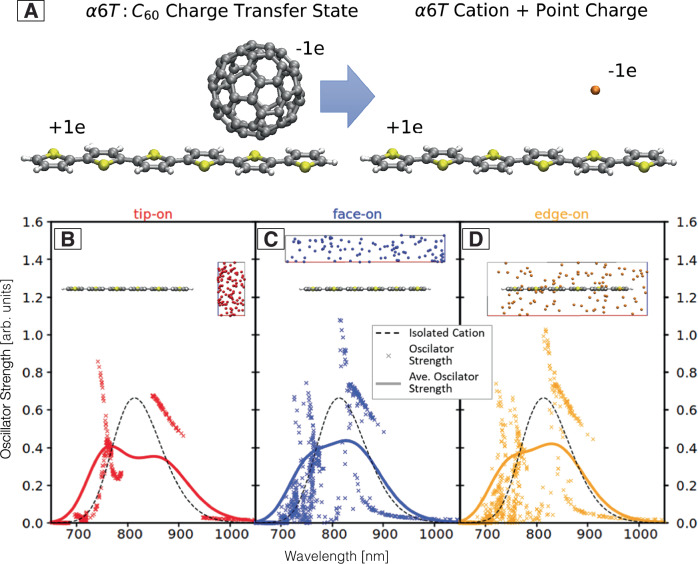
Absorption spectra simulated with the minimal CT model. **A** Minimal CT model used to simulate the absorption spectrum of the α6T cation in vicinity of the C_60_ anion. The quasi-spherical C_60_ anion is replaced by a negative point charge positioned at the centre of the C_60_ molecule. Wavelengths and oscillator strengths for optical transitions in tip-on (**B**), face-on (**C**), and edge-on (**D**) position compared to the isolated cation (black dashed line) as obtained from TD-DFT/B3LYP/6−31 G* and corrected by a rigid offset (red shift by 0.47 eV). Symbols represent optical transitions for individual point charge positions, lines represent the average over 100 different CT orientations convoluted with Gaussians of 80 meV width.

Point charge positions were chosen randomly within defined regions to include different face-on, tip-on, or edge-on orientations, i.e., addressing scenario (i) (Fig. 3). In accordance with the similar experimental α6T cation spectrum between the ‘dilute-donor’ and bilayer samples (comparable broadening and shift, Figs. S3–S5), we do not consider different bulk and isolated sub-species of charges. On the one hand, not considering bulk phase α6T for the dilute blends studied here is justified by the fact that α6T molecules are isolated at dilutions of less than 10%^8,24,26^. The similar cation spectrum in the bilayer suggests that charges do not delocalize in the α6T layer either, which might be related to the particular processing and molecular packing in the evaporated films. On the other hand, we do not need to consider a distinction of SC cations due to different local dielectrics, because the possible influence on transition energies (in the order of 5 meV)^37^ is smaller than our applied Gauss broadening for the transitions (80 meV) used to simulate the TA spectra. Thus, we only simulate the TAS signature of the α6T cation either alone (SC) or with a counter charge in its vicinity (CTC). In accordance with Fig. S8, the C_60_ is not explicitly considered, as the corresponding absorption (at about 1070 nm or 1.15 eV) is far enough from the one of the α6T cation not to have an effect^30^.

For a representative absorption study of the α6T cation in the CT state, it is important to densely sample different possible CT state arrangements. The dominating orientations are different for different degrees of phase separation in the blends. While for the bilayer structure the interface is predominantly formed by tip-on configurations with C_60_ on top of the α6T herringbone structure (Fig. 2A)^34^, in blends with low α6T content, face-on or edge-on arrangements become equally likely. Figure 3A shows our minimal model of the CT states by placing a negative point charge, which represents the C_60_ anion, near the α6T cation. To sample different orientations, we simulate different classes of geometries of the anionic C_60_ relative to a6T: face-on, tip-on, edge-on, and finally complete surrounding, with the resulting spectra summarized in Fig. 3B–D. We find that the transition energies and oscillator strengths depend only weakly on the location of the negative point charge. We observe a bimodal distribution of transition energies, leading to a broad band via convolution of all points (solid lines). This characteristic bimodal band is found for all classes (face-on, tip-on or edge-on) and is thus representative of the absorption of CTC cations independently of the relative orientation between molecules. It therefore represents both bilayer and BHJ films. From the universal characteristics, we can therefore exclude scenario (i), i.e., that the spectral differences of C1 and C2 in the TA data are related to the molecular arrangement between the anion and cation.

**Fig. 4:**
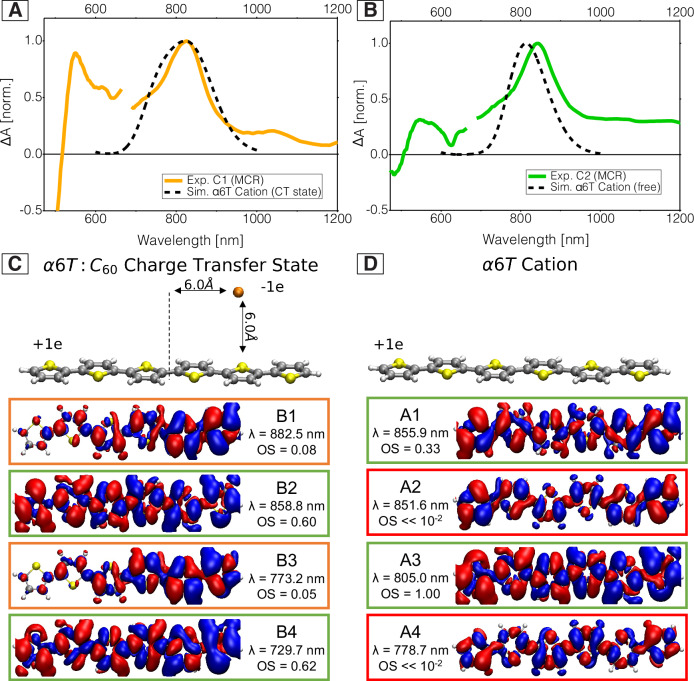
Simulated spectra and isosurface plots of transition densities. **A** Simulated absorption of the α6T cation from TD-DFT in a CTC configuration together with the experimental C1 component from the MCR-ALS analysis of the 5% ‘dilute-donor’ blend (noise around 610 nm is due to pump scattering). **B** Simulated absorption of the α6T cation in SC configuration together with the experimental C2 component. **C** Isosurface plots of transition densities, resulting wavelengths and oscillator strengths (OS) as obtained from TD-DFT/B3LYP/6−31 G* and corrected by a 0.47 eV red shift for the CTC cation transitions (B1-4) with a negative point charge in face-on position at distance of 6 Å and a lateral shift of 6 Å in the conjugated backbone direction, and (**D**) for the separated α6T cation transitions (A1-4).

Considering scenario (ii), the spectrum obtained by averaging over point charge positions surrounding the α6T molecule is representative for the CTC cation absorption and is plotted in Fig. 4A together with the experimental MCR component of C1. It is clearly different from the calculated cationic absorption in neutral environment, i.e., the SC cation, shown in Fig. 4B with the C2 component. The absorption spectrum of the separate α6T cation has a maximum at 1.53 eV (810 nm) that involves two transitions (*vide infra*). In contrast, the spectrum with a negative counter charge is broader due to an additional shoulder on the blue side of the spectrum at about 1.65 eV (750 nm). Hence, the α6T cation in the CT state appears strongly blue shifted with respect to the separate α6T cation. In Fig. 4A, B, we therefore find a reasonable agreement in spectral shape between the first charge component (C1) with the simulated CTC spectrum and the second charge component (C2) with the simulated SC spectrum. The two charge species C1 and C2 are therefore attributed to CTCs and SCs, respectively, rationalizing the shift and the narrowing of the cation band in the TA spectra. Note that this conclusion is also consistent with the reduced EA signature in the C2 component (weaker electric field when the charges are far apart) and with the broader C_60_ anion band for the SCs due to higher delocalization of the separated anions into C_60_ clusters. Previously, we have reported shifts in the EA signature during charge separation in dilute blends based on the 1,1-Bis[(di-4-tolylamino)phenyl]cyclohexane (known as TAPC) donor, which do not occur here because of different local electrostatic interactions between α6T and C_60_^6^.

The physical origin of the spectral change between the CTC and SC cation is studied in more detail by analysing the contributing optical transitions. Figure 4C, D display the transition densities for the transitions in the range between 700 nm and 860 nm for a representative CTC model with the point charge at a face-on position, and for the SC model. The absorption of the separate α6T cation in this spectral region is dominated by the transitions that are indicated as A1 and A3, causing the dominant absorption (A3) at about 800 nm with the shoulder (A1) at about 855 nm. In the presence of the negative counter charge, the stronger A3 transition splits and gives rise to transitions B2 and B4, which causes the blue shift and the bimodal shape in the spectral signature of the CTC cation. The weakly active B1 and B3 transitions clearly exhibit the strong symmetry break that is induced by the point charge. They are formed by the former active transition A1 with contributions of A2 or A4, respectively, which becomes clear when comparing the shapes of the isosurfaces of the transition densities, in particular on the outmost thiophene rings. This results in the confinement for the transition densities of B1 and B3. The symmetry break also affects the A3 transition, which originally shows an antisymmetric transition density upon 180° rotation around the axis normal to the molecular plane. This symmetry is lost in the derived B2 and B4 transitions, which also inherit the original oscillator strength of A3. We conclude that the broken molecular symmetry caused by the C_60_ anion monopole charge results in a splitting of the optical transition peaks and consequently in the observed bimodal distribution of transition energies.

### Kinetic modelling of the photoinduced processes in α6T:C_60_ systems

**Fig. 5:**
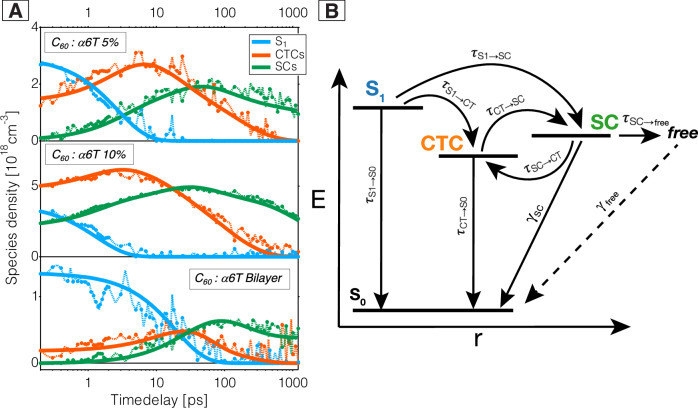
Kinetic modelling of the exciton and charge dynamics. **A** Dynamics of the S_1_ excitons (blue), CTCs (orange) and SCs (green) for the 5% blend (top, 4.4 × 10^18^ cm^3^ excitation density), the 10% blend (middle, 1.2 × 10^19^ cm^3^) and the α6T:C_60_ bilayer (bottom, 1.5 × 10^18^ cm^3^) as determined by MCR-ALS decomposition, together with the fits obtained from the kinetic modelling. **B** Jablonski diagram summarizing the relaxation pathways in the samples, used as the basis for the kinetic model.

Knowing from the theoretical calculations that the C1 and C2 species seen in the experimental TA spectra correspond to CTCs and SCs, respectively, we now exploit this to gain unique physical insight about the charge separation mechanism. A kinetic model is designed to follow the sequence of processes undergone upon excitation of the ‘dilute-donor’ blends (5% and 10%) and the bilayer. The kinetic model is then fit to the dynamics obtained from the MCR-ALS decomposition of the TA spectra in order to quantify the time constants associated with each step. As seen in Fig. S9, the dynamics from different spectrometers (VIS, NIR1 and NIR2) and during several measurement series are consistent, showing clear trends with excitation density (no dependence for excitons and CTCs, increased recombination for SCs). Therefore, all data for each sample were analysed globally with the kinetic model, yielding excellent fits (Figs. S10–S12). Figure 5A depicts the fit of the model to the species dynamics at selected excitation densities, while Fig. 5B summarizes the Jablonski diagram describing the kinetic model. The Jablonski diagram is expressed as a set of linked differential equations (Eqs. 1–4) for each species population. Equation 1 is fit to the C_60_ exciton (S_1_) dynamics, Eq. 2 to the CTC dynamics and the sum of Eqs. 3 and 4 to the SC dynamics. Due to our limited experimental time window of 1–1.5 ns, the recombination of long-lived SCs (i.e., ‘free’ SCs) is not observed and they appear as a plateau in the experimental data at low excitation densities. To model this, a spectrally undistinguishable sub-population of ‘free’ SCs ( = Fs) is needed (Eq. 4), while Eq. 3 describes the SCs undergoing fast recombination on the investigated nanosecond time scale.

1 $$\frac{{{\rm{d}}}{S}_{1}}{{{\rm{d}}}t}=-{\tau }_{S1\to S0}^{-1}{\left[{S}_{1}(t)\right]-\tau }_{S1\to {CT}}^{-1}\left[{S}_{1}(t)\right]-{\tau }_{S1\to {SC}}^{-1}\left[{S}_{1}(t)\right]$$

2 $$\frac{{{\rm{d}}}{CTC}}{{{\rm{d}}}t}={\tau }_{S1\to {CT}}^{-1}\left[{S}_{1}(t)\right]-{\tau }_{{CT}\to S0}^{-1}\left[{CTC}\left(t\right)\right]-{\tau }_{{CT}\to {SC}}^{-1}\left[{CTC}\left(t\right)\right]+{\tau }_{{SC}\to {CT}}^{-1}\left[{SC}\left(t\right)\right]$$

3 $$\frac{{{\rm{d}}}{SC}}{{{\rm{d}}}t}={\tau }_{S1\to {SC}}^{-1}\left[{S}_{1}(t)\right]+{\tau }_{{CT}\to {SC}}^{-1}\left[{CTC}\left(t\right)\right]-{\tau }_{{SC}\to {CT}}^{-1}\left[{SC}\left(t\right)\right]-{\tau }_{{SC}\to {free}}^{-1}\left[{SC}\left(t\right)\right]-{\gamma }_{{SC}}^{-1}{\left[{SC}\left(t\right)\right]}^{2}$$

4 $$\frac{{{\rm{d}}}F}{{{\rm{d}}}t}={\tau }_{{SC}\to {free}}^{-1}\left[{SC}(t)\right]-{\gamma }_{{free}}^{-1}{\left[F\left(t\right)\right]}^{2}$$

Table 1 reports the resulting time constants from the kinetic modelling. The excitons in the S_1_ state can either intrinsically decay to the ground state (S_0_) with a time constant of 150 ps, as determined by previous studies on neat C_60_ films^30^. Most of them are however quenched in the presence of the α6T donor, if they are generated close to a donor molecule (prompt HT), or if they are able to diffuse to an α6T:C_60_ interface within their lifetime (diffusion-limited HT). There, they undergo HT to form CTCs or directly SCs^38^, with time constants τ_S1→CT_ and τ_S1→SC_, respectively. We note that, following the convention used in the field, we refer to the HT times after photoexcitation, which includes both the diffusion and intrinsic charge transfer steps. Judging from the ratio between the time constants, only a negligible fraction of the excitons in the ‘dilute-donor’ blends decays to the S_0_ state (1–2%), which increases to 13% in the more phase-separated bilayer. At 0.2 ps, there are some promptly generated initial charges in the 10% blend (63% CTCs and 13% SCs), in the 5% blend (40% CTCs and 0% SCs) and in the bilayer (10% CTCs and 0% SCs), depending on the probability of forming excitons in the proximity of α6T molecules. The fact that SCs are generated promptly in the 10% blend confirms their direct generation pathway from S_1_ excitons. Direct SC generation has been reported before in other fullerene-based systems and is likely related to delocalization into C_60_ clusters^14,39,40^.

**Table 1:** Outcome of the kinetic modelling of the MCR-ALS decomposition dynamics from the TA data for the dilute α6T:C_60_ blends (5% and 10%) and the bilayer

	α6T:C_60_ 5%	α6T:C60 10%	α6T:C60 Bilayer
^a^Exciton_initial_	~ 60%	~ 30%	~ 90%
^a^CTC_initial_	~ 40%	~ 50%	~ 10%
^a^SC_initial_	~ 0%	~ 20%	~ 0%
${}^{{{\rm{b}}}}{{\rm{\tau }}}_{{{\rm{S}}}1\to {{\rm{S}}}0}$ (ps)	150 (2%)	150 (1%)	150 (13%)
${}^{{{\rm{c}}}}{{\rm{\tau }}}_{{{\rm{S}}}1\to {{\rm{CT}}}}$ (ps)	4.3 (63%)	2.4 (55%)	36 (53%)
${}^{{{\rm{c}}}}{{\rm{\tau }}}_{{{\rm{S}}}1\to {{\rm{SC}}}}$ (ps)	7.7 (35%)	3.0 (44%)	55 (35%)
${}^{{{\rm{d}}}}{{\rm{\tau }}}_{{{\rm{CT}}}\to {{\rm{S}}}0}$ (ps)	197	197	72
${}^{{{\rm{e}}}}{{\rm{\tau }}}_{{{\rm{CT}}}\to {{\rm{SC}}}}$ (ps)	38	29	79
${}^{{{\rm{e}}}}{{\rm{\tau }}}_{{{\rm{SC}}}\to {{\rm{CT}}}}$ (ps)	50	40	157
${}^{{{\rm{f}}}}{{\rm{\tau }}}_{{{\rm{SC}}}\to {{\rm{free}}}}$ (ps)	370	195	384
${}^{{{\rm{g}}}}\gamma _{{{\rm{SC}}}}$ (ps·cm^-^^3^)	4 × 10^20^	6 × 10^20^	2 × 10^22^
${}^{{{\rm{g}}}}\gamma _{{{\rm{free}}}}$ (ps·cm^-3^)	7 × 10^21^	7 × 10^21^	3 × 10^22^

^a^Initial population of excitons (Exciton_initial_) and of both charge species (CTC_initial_, SC_initial_) given at 0.2 ps (experimental time resolution) and expressed as a fraction of the total excitation density;

^b^Intrinsic exciton lifetime (τ_S1→S0_) and the fraction of the initial exciton population (at 0.2 ps) decaying due to exciton recombination to the ground state;

^c^Hole-transfer (HT) time constants (τ_S1→CT_, τ_S1→SC_) to both charge species along with the fraction of initial excitons undergoing HT to each;

^d^Charge recombination time constant of CTCs (τ_CT→S0_);

^e^Time constants associated with charges separating or undergoing fast re-encounter, respectively (τ_CT→SC_, τ_SC→CT_);

^f^Time constant associated with SCs remaining free at long time delays (τ_SC→free_);

^g^Bimolecular time constant (reciprocal of rate) for the recombination of SCs and ‘free’ SCs (*γ*_SC_, *γ*_free_).

Concerning the diffusion-limited HT (quantified by τ_S1→CT_ and τ_S1→SC_,), this is fastest in the 10% blend, with HT to CTCs having a time constant of 2.4 ps and HT to SCs of 3.0 ps, as opposed to the 5% blend where HT to CTCs has a time constant of 4.3 ps and HT to SCs of 7.7 ps. Due to the increased separation between donor and acceptor in the bilayer, this slows down further to 36 ps and 55 ps, respectively. The trend agrees with a diffusion-controlled HT model where excitons require, on average, a shorter distance to reach a donor molecule in the 10% blend^6,7,30,41–44^. Comparing τ_S1→CT_ with τ_S1→SC_, we note that the SCs are always formed slightly slower, but both time constants are on the same order of magnitude. We conclude that CTCs and SCs are generated from the excitons in α6T:C_60_ in approximately equal amounts.

Once CTCs are generated, they either recombine to the ground state or separate reversibly to SCs (Fig. 5B). The equal CT recombination time of τ_CT→S0_ = 197 ps for both 5% and 10% blends is due to their similar CT energy levels^17,24^, while the faster recombination in the bilayer (72 ps) points to a lower-lying CT state due to different interfacial energetics and donor aggregation (Table 1). The CTC dissociation to SCs is comparable for both blends (38 ps in 5% and 29 ps in 10%) and slower in the bilayer (79 ps), which is likely related to the local dielectric environment and electron delocalization into C_60_ clusters. Due to monomolecular back-transfer of SCs to CTCs, there is an equilibrium between the more or less bound charges, which is significantly more shifted towards SCs in the bilayer (τ_SC→CT_ = 157 ps, twice as slow as τ_CT→SC_). The enhanced SC to CTC back-transfer in the ‘dilute-donor’ blends is caused by the dispersion of the donor molecules. In fullerene-based ‘dilute-donor’ solar cells, hole transport has been shown to occur by long range tunnelling from one α6T molecule to the next^24^. However, over the 1.5 ns time-window of our TA measurement, the holes are essentially immobile, while the electron mobility in C_60_ remains unperturbed by the α6T molecules for blends ≤10%^24^. Thus, holes that are associated with SCs are trapped on α6T molecules, while electrons are mobile in the C_60_ clusters. This can lead to fast, trap-based recombination, when the electrons re-encounter trapped holes and re-form CTCs that recombine to the ground state^6^. CT state re-formation is slightly faster in the 10% blend than the 5% blend (40 ps and 50 ps, respectively) owing to the increase in α6T site density. In the dilute blends, the re-encounter of SCs to CTCs occurs on a similar time scale as the CTC dissociation to SCs, as both processes are dominated by the electron mobility. While dominating in thin films, the trap-based recombination is suppressed in a device configuration by the fast extraction of mobile electrons, explaining the high OPV efficiency at low donor concentrations^45^.

At higher excitation densities, we notice a faster decay of SCs in the 5% and 10% blends, leading to an overall lower SC yield even at short time delays (Figs. 1B and S9). This is due to bimolecular recombination of the SCs, which occurs with a bimolecular recombination time constant of ~ 4-6x10^20^ ps·cm^-^^3^ (Table 1). Since our experimental time window is limited to 1.5 ns, we cannot follow the recombination of all SCs and see some of them (called ‘free’ SCs) as a long-lived plateau in the dynamics (Fig. 5A). The ‘free’ SCs are formed within hundreds of picoseconds, with τ_SC→free_ having a large uncertainty due to their undistinguishable spectral signature. As they are not extracted in the film, the ‘free SCs’ eventually decay with a bimolecular time constant of 7 × 10^21^ ps·cm^-3^ (only noticeable at the highest fluences in our experimental conditions). In the bilayer, we do not reach a regime where bimolecular recombination is observed (Figs. S9 and S12), first because the achieved charge density is lower and second, because the phase separation reduces the encounter of electrons and holes, leading to much slower bimolecular recombination time constants of 2–3 × 10^22^ ps·cm^-3^ for both SCs and ‘free’ SCs (Table 1).

## Discussion

In summary, α6T:C_60_ systems in bilayer and ‘dilute-donor’ morphologies were experimentally investigated using TAS. The phase-separated bilayer as well as the 5% and 10% dilute blends exhibited important charge generation. Moreover, in all samples, the α6T cation peak absorption showed a definite and measurable red shift and narrowing over time. A spectral decomposition (MCR-ALS) was performed which resulted in an excitonic spectrum and two distinguishable charge components, whose distinct dynamics caused the spectral changes. TD-DFT simulations were undertaken to simulate the α6T cation transition energies in the vicinity of the C_60_ anion and in isolated α6T, representing the hole in the CT state and the separated hole, respectively. In the presence of the C_60_ anion, the charge causes an electric field that breaks the molecular symmetry of α6T molecules in close proximity and influences their optical selection rules. The simulated spectra were in good agreement with the two charge components found in the TAS data. It was therefore concluded that one charge species is due to CTCs at the donor:acceptor interface and the other to SCs. Such proximity-induced symmetry-breaking can be expected in highly symmetric molecules, such as the rod-like α6T. Moreover, resolving the related spectral differences requires limited conformational disorder, which is possible in small molecule systems but not in polymeric donors, where different conformations and chromophore sizes co-exist. Thus, our findings constitute the first direct and unambiguous visualization of spectrally resolved CTCs and SCs. This allowed the formulation of a complete and nuanced kinetic model to quantify the interconversion processes and decay dynamics of the exciton and the charge species with unique insight to the charge generation, separation, and recombination mechanisms.

Thermally evaporated OPVs represent a commercially viable technology currently leading the organic solar cell market^23^. Therefore, the mechanistic details that we unravel here are of high significance to understanding functional devices. We demonstrate that SCs are formed in two ways in equal amounts: directly from the excitons within a few picoseconds or more slowly (~30–80 ps) from reversible splitting of CTCs. This resolves a long-standing debate about which of the two processes generates photocurrent in OPVs. The spectral differences between CTCs and SCs were seen in both the ‘dilute-donor’ and the bilayer samples, showing that the phenomena are not specific to an intermixed or phase-separated morphology. Our findings can be generalized to solution-processed polymer:acceptor blends. Here, CTCs and SCs have not been distinguished by TAS so far, so that the CT state dynamics has only been indirectly speculated, leading to contradictory reports whether excitons dissociate directly to SCs (by coupling into delocalized fullerene clusters, via hot processes or by long-range electron transfer)^10,11,14,19–21,38,46–50^, or whether relaxed CTCs are generated first and subsequently split^12,15,17,18,46,51^. We show that both processes can co-exist and have successfully applied a similar kinetic model as the one presented here to describe the charge generation dynamics in a solution-processed polymer:fullerene system with low S_1_-CT offset^15^. Without possibility to directly distinguish CTCs and SCs, we have used electro-modulated differential absorption (EDA) spectroscopy to verify the modelled dynamics of the SCs. Another possibility to estimate the SC formation rate is from the short-range charge mobility obtained from terahertz (THz) measurements^13,52^.

Ongoing work in our group indicates that the mechanisms demonstrated here are equally relevant for blends containing non-fullerene acceptors (NFAs), which benefit from high Voc and PCEs exceeding 18%^5,53^. Here, fullerene clusters are absent and higher-lying CT states are energetically inaccessible due to low S_1_-CT driving force, so that SCs are mainly generated by the slower route of CTC dissociation^15,16,54–57^. Our current study highlights the relevance of the kinetic competition between this charge separation and CTC re-formation/recombination, which ultimately determines whether charges are lost or extracted as current. For the ‘dilute-donor’ thin films, the competing recombination channel is predominantly trap-based due to the quasi-immobile holes. However, since all charge recombination proceeds via the CT state^58–62^, its splitting, re-formation and decay dynamics is equally relevant to geminate and bimolecular recombination processes occurring over a wide range of time scales^63–66^. We, therefore, stress the importance of either identifying subtle spectral changes between CTCs and SCs using theoretical methodology or, in the absence of distinct absorption characteristics, to include charge separation over several length scales (from CSCs to SCs to F) when kinetically modelling ultrafast data of modern NFA-based OPVs.

## Methods

### Sample preparation

α-Sexithiophene (α6T) was purchased from Lumtec Corp. once sublimed. C_60_ was purchased from Creaphys GmbH with >99.99% purity. Films fabricated for transient absorption measurements were processed on glass (Eagle XG, Thin Film Devices Inc). Substrates were cleaned by sequential sonication in detergent (Hellmanex GmbH), de-ionized water (DI), acetone and isopropanol (IPA) at 55 °C for 10 min each and finally UV-ozone treated for 10 min. Thin films were evaporated onto the substrate in a custom deposition tool (Creaphys GmbH) and transferred to a nitrogen-filled glove box without air exposure. The substrate was held at room temperature during deposition. The deposition rate was controlled by quartz crystal microbalances calibrated by X-ray reflectivity measurements with ellipsometry measurements for thickness (50 nm). For the blended films, the relative deposition rates of the materials were varied to obtain the desired α6T:C_60_ ratio. All ratios are given in molar percent. The samples were kept in a nitrogen atmosphere and in the dark to avoid degradation and C_60_ dimerization.

### Transient absorption spectroscopy (VIS/NIR1)

The transient absorption spectroscopy (TAS) experiments were carried out using inhouse built setups using the output from a regeneratively amplified Ti:sapphire laser system (Astrella from Coherent, 1 kHz, 6 mJ, 800 nm pulse). The output of the Astrella is split into two beams in order to generate both the pump and probe from the same pulse. For experiments probing in the visible (VIS) and 820–1200 nm range (NIR1), the pump beam was frequency-converted from 800 nm to 610 nm using a commercial optical parametric amplifier (OPerA Solo, Coherent). The broad ‘white-light’ probe beam was generated by passing the second part of the fundamental 800 nm beam through a 5 mm thick sapphire crystal generating a continuum from 450 nm to 1200 nm. With the white light generation being inhomogeneous, a relatively large intensity around 800 nm remains in the probe beam. In order to remain sensitive to the shorter and longer wavelengths, the 800 nm part of the continuum was removed, and the visible and near-IR ranges measured separately. The VIS range was isolated using a 720 nm low pass filter and NIR1 using a 850 nm high pass filter, resulting in an unavoidable gap around 800 nm. The probe beam was split before the sample into a reference beam (to correct for laser intensity fluctuations) and a signal beam. The latter was then focused on the sample where it overlapped spatially and temporally with the pump pulses at a magic angle polarization difference. The probe intensity was negligible with respect to the pump intensity with an energy (below 5nJ over a diameter of 180 μm). The temporal delay between the pump and probe beams was achieved by varying the optical pathlength of the probe pulses with respect to the pump pulses using a computer-controlled delay stage (up to 1.5 ns). The VIS and NIR1 were recorded separately with two spectrographs (assembled by Entwicklungsbüro Stresing, Berlin), consisting each of a home-built prism spectrometer equipped with either two 512 × 58 pixel back-thinned Silicon CCDs (Hamamatsu S07030- 0906 for VIS) or with two InGaAs arrays (Hamamatsu for NIR1), for both the signal and reference beams. The pump pulses were chopped at 500 Hz and the probe pulses were recorded shot-by-shot (1 kHz). The spectra at each time step were measured 32,000–80,000 times (4000 shots over 8–20 scans) and subsequently averaged to achieve the best signal-to-noise. Thermal effects in the TA spectra were excluded, since the investigated film absorbance showed only a weak temperature dependence below 500 nm, thus outside the TAS window (Fig. S13).

### Transient absorption spectroscopy (NIR2)

For broad-band detection from 690–1600 nm (NIR2), the 800 nm laser output (Astrella from Coherent, 1 kHz, 6 mJ, 800 nm pulse) generated a signal at 1300 nm and an idler at 2100 nm in a home-built two-pass optical parametric amplifier (OPA). The idler was then used as a seed for white-light generation in a YAG crystal. For the pump path, the 800 nm pulse was used to generate a 610 nm excitation pulse in a home-built NOPA (the achievable pulse energy was much lower than with the OPA in the VIS/NIR1 setup, so that the beam had to be more focused to reach high excitation densities). The pump was chopped at 500 Hz and the intensity of the probe was recorded on an Entwicklungsbüro Stresing camera with InGaAs pixels (Hamamatsu) with detection from 400–1700 nm.

### Multivariate curve resolution with alternating least squares (MCR-ALS)

Prior to analysis, all TA spectra were corrected for the chirp in the probe pulses as well as for the background, by subtracting the spectrum at pre-pump timedelays. MCR-ALS is a soft modelling technique used to factor out the TA spectra into a limited number of components and the dynamics of these components in time:

5 $$D=C{S}^{T}+E$$

where **D** is the TA data matrix, **C** the temporal dynamics, **S** the spectral components and **E** the residual matrix. The temporal dynamics and shape of spectral components are then optimized such that **E** is minimized. The decomposition is done using the pyMCR python package^67^. The number of components is determined to be three (one exciton and two charge species, Fig. S6) and spectral or dynamic guesses are given to initialize the fitting. The exciton component is taken from the early time TA spectrum of a neat C_60_ film and scaled according to the number of absorbed photons and film thickness, yielding the extinction coefficient. Since the amplitude and shape of the exciton species is well known, this component is held constant throughout the MCR-ALS fitting and allowed to determine the density of excitons and their temporal evolution for each measurement. A unimodality constraint was placed on the exciton dynamics as we do not expect meaningful back-transfer from the charge species to the exciton. On the other hand, the two charge components were optimized during the MCR fitting for each type of sample. In the 5% blend, only excitons and CTCs are present at early times (0.2–1.0 ps), so that subtracting the exciton density from the total excitation density allowed to find the density and extinction coefficient of the CTCs. A similar procedure, subtracting both the exciton and CTC populations from the total excitation density in the 10% blend then yielded the extinction coefficient of the SCs. This was done at all fluences simultaneously to increase the robustness of the result.

### Density functional theory (DFT) calculations

Molecular models of α6T cations were studied using DFT as implemented in the Computational Chemistry Package NWChem 6.8^68^. All calculations were performed with the hybrid B3LYP exchange-correlation functional and the 6–31 G* basis set^69,70^. After structure optimization, the optical transitions of separated charges and charge transfer state charges were determined using time-dependent density functional theory (TD-DFT)^71–73^. Charge transfer states were modelled by placing a negative point charge next to the α6T cation representing the C_60_ anion (see Fig. 3A of the main text). To test this minimal model, the negative C_60_ was considered explicitly for a single orientation and a smaller basis set (see Fig. S8), using constrained DFT (cDFT) to localise positive and negative charge on α6T and C_60_, respectively. The obtained transition energies were corrected by a rigid red shift of 0.47 eV (0.55 eV for cDFT) to include solid state effects phenomenologically in the gas phase model and to match the experimentally observed absorption at 650–900 nm. Simulated absorption spectra were obtained by superposition of Gauss functions with width 80 meV scaled by the individual oscillator strengths of each transition.

### Kinetic modelling

The interconversion of the light-induced species (determined by MCR-ALS) was fit to a kinetic model consisting of a series of linked differential equations (shown by Eqs. 1–4 above) derived from the Jablonski diagram (Fig. 5B). The model was fit for each time constant with only the exciton lifetime held fixed as it was already known^30^. The model was fit using the differential evolution minimiser with a custom least squares loss function where the separated and free charge populations were summed before being compared against the second charge component. The fit was done using the symfit python package^74^.

## Supplementary information

Supplementary Information

## Data Availability

The data that support the findings of this work are available as open access in the BORIS Repository of the University of Bern at https://boris-portal.unibe.ch/handle/20.500.12422/66368. Additional raw data can be obtained from the authors upon request.
