# Supplementary material for: Direct visualization of the charge transfer state dynamics in dilute-donor organic photovoltaic blends
Source: Nat Commun. 2024 Nov 14;15:9851. doi: 10.1038/s41467-024-53694-4 (PMC11561185; doi:10.1038/s41467-024-53694-4)
Supplement: Supplementary file 1 — Supplementary Information [file 41467_2024_53694_MOESM1_ESM.pdf]

Supplementary Information

Direct Visualization of the Charge Transfer State Dynamics in Dilute-Donor Organic Photovoltaic Blends

Gareth John Moore[1,†] Florian Günther[2,3,4,†] Kaila M. Yallum[1], Martina Causa'[1], Anna Jungbluth[5], Julien Réhault[1], Moritz Riede[5], Frank Ortmann[4,6]*, Natalie Banerji[1]*

†These authors contributed equally.

[1]Department of Chemistry, Biochemistry and Pharmaceutical Sciences, University of Bern, Freiestrasse 3, 3012 Bern, Switzerland.

[2]Instituto de Física de São Carlos (IFSC), Universidade de São Paulo (USP), Av. Trabalhador saocarlense, 400, CEP 13560-970 São Carlos, Brazil

[3]Instituto de Geociências e Ciências Exatas (IGCE), São Paulo State University (UNESP), Jerdim Bela Vista, Rio Claro, CEP 13506-900, Brazil.

[4]Center for Advancing Electronics Dresden, Technische Universität, Helmholtzstraße 18, 01069 Dresden, Germany.

[5]Clarendon Laboratory, Department of Physics, University of Oxford, Parks Road, OX1 3PU, Oxford, United Kingdom.

[6]TUM School of Natural Sciences, Department of Chemistry, Technische Universität München, Lichtenbergstrasse 4/V, 85748 Garching b. München, Germany.

Corresponding Authors

natalie.banerji@unibe.ch, frank.ortmann@tum.de

Supplementary Figures

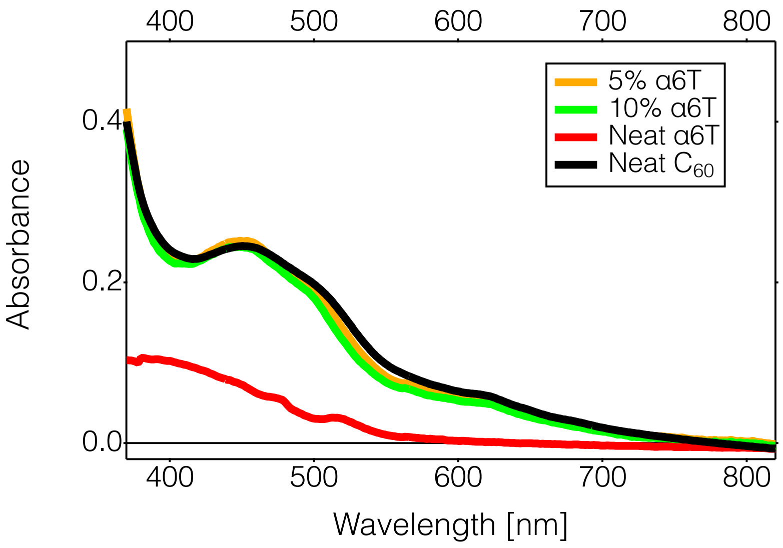

Supplementary Figure 1. Absorbance spectra of the investigated thin films. Steady-state absorbance spectra of 50 nm thin films of 5% and 10% α6T:C_{60} together with films of neat C_{60} and neat α6T. The latter absorbs below 550 nm and hardly affects the blend absorbance due to its low concentration.

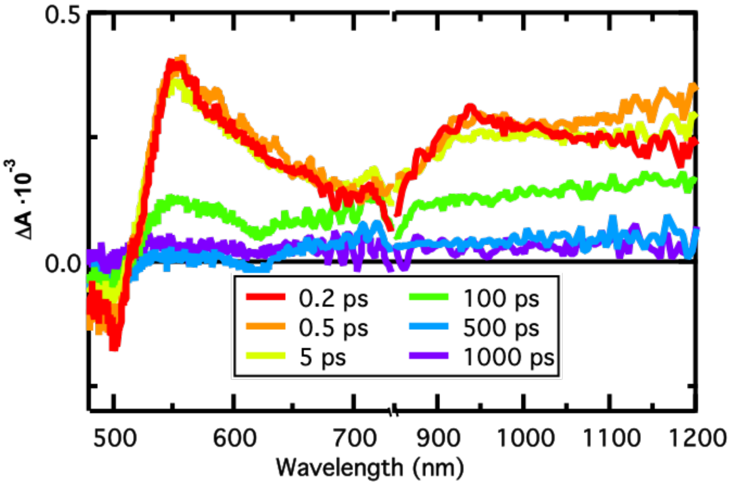

Supplementary Figure 2. Transient absorption spectra of a neat C_{60} film. TA spectra of a neat C_{60} thin film (50 nm) at time delays of 0.2 ps, 0.5 ps, 5 ps, 100 ps, 500 ps, and 1000 ps are shown. The neat film was excited at 610 nm with an excitation density of $6.2x10^{18}$ cm^3, such that only Frenkel type excitons are generated.[1, 2] The exciton signature consists of a negative ground state bleaching around 500 nm and excited state absorption at 550 nm and 930 nm. The exciton signature decays with a time constant of 150 ps.

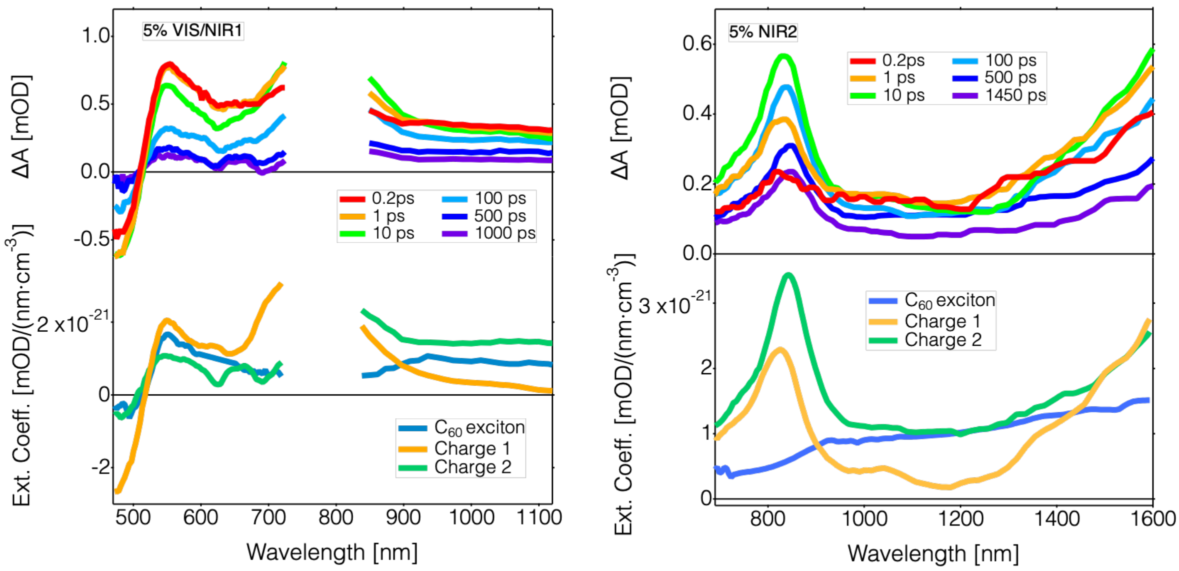

Supplementary Figure 3. Transient absorption data of the 5% sample. Top panel: TA spectra of the 5% α6T:C_{60} blend at selected time delays (0.2 ps, 1 ps, 10 ps, 100 ps, 500 ps, and 1.0-1.5 ns) with 610 nm pump excitation at a density of 1.0x10^{19} cm^{-3} (VIS/NIR1) or 4.4x10^{18} cm^{-3} (NIR2). Bottom panel: Spectral components of the MCR-ALS decomposition obtained for all fluences measured for the 5% blend, with the C_{60} Frenkel-type exciton (blue, held constant as in neat C_{60} film), the first charge component (orange, Charge 1) and the second charge component (green, Charge 2), expressed as the extinction coefficient of the three species.

Comment: TA spectra of the investigated samples (5%, 10% and bilayer) measured with the VIS, NIR1 and NIR2 spectrometers after excitation at 610 nm (at one of the used fluences) are shown in **Supplementary Figures 3-5**. Three different spectrometers were used to cover different spectral regions and show consistent results (VIS: 470-720nm, NIR1: 840-1150nm, NIR2: 690-1600 nm). The lower panels show the species components of excitons and charges obtained by MCR-ALS analysis. The extinction coefficients were determined from the excitation density, as described in the methods section.

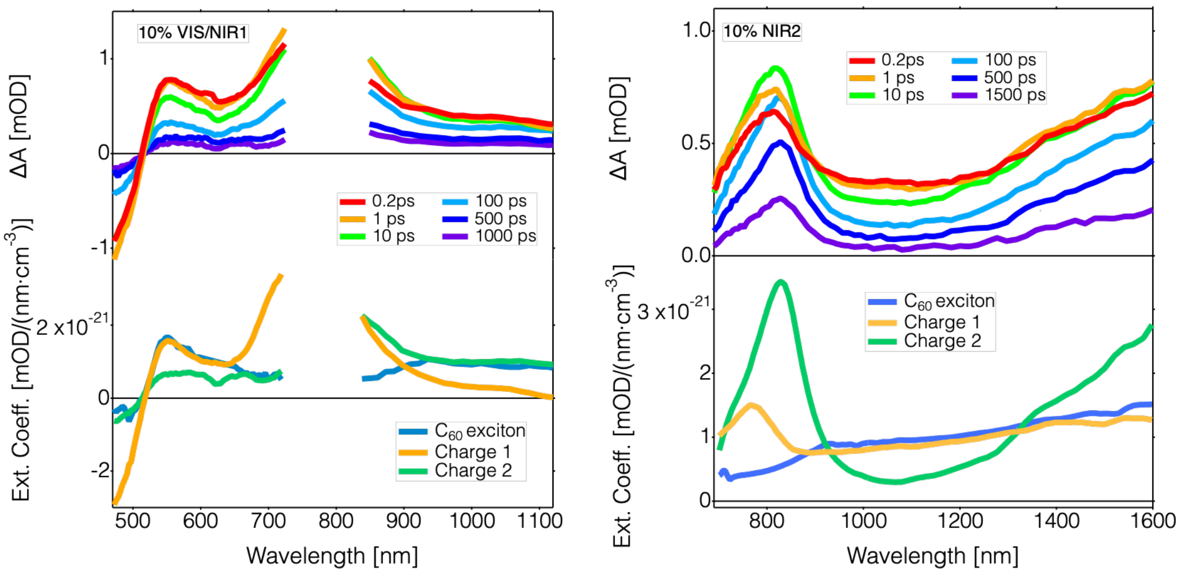

Supplementary Figure 4. Transient absorption data of the 10% sample. Top panel: TA spectra of the 10% α6T:C_{60} blend at selected time delays (0.2 ps, 1 ps, 10 ps, 100 ps, 500 ps, and 1.0-1.5 ns) with 610 nm pump excitation at a density of $1.2x10^{19}$ cm^{-3} (VIS/NIR1) or $8.6x10^{18}$ cm^{-3} (NIR2). Bottom panel: Spectral components of the MCR-ALS decomposition obtained for all fluences measured for the 10% blend, with the C_{60} Frenkel-type exciton (blue, held constant as in neat C_{60} film), the first charge component (orange, Charge 1) and the second charge component (green, Charge 2), expressed as the extinction coefficient of the three species.

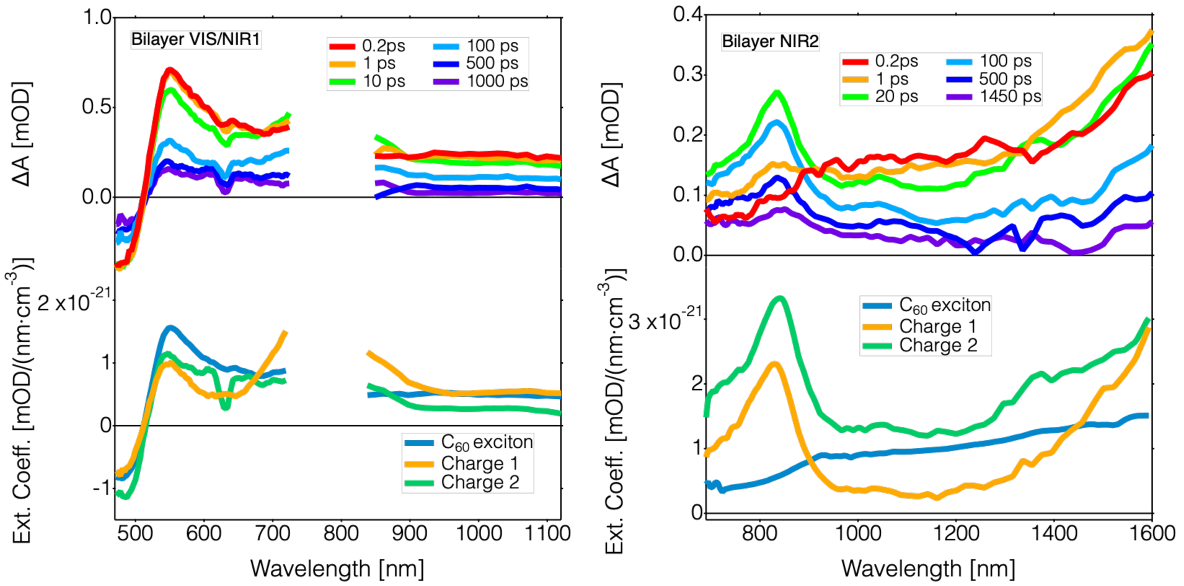

Supplementary Figure 5. Transient absorption data of the bilayer sample. Top panel: TA spectra of the α6T:C_{60} bilayer at selected time delays (0.2 ps, 1 ps, 10 ps, 100 ps, 500 ps, and 1.0-1.5 ns) with 610 nm pump excitation at a density of $1.0x10^{19}$ cm^{-3} (VIS/NIR1) or $1.5x10^{18}$ cm^{-3} (NIR2). Bottom panel: Spectral components of the MCR-ALS decomposition obtained for all fluences measured for the bilayer, with the C_{60} Frenkel-type exciton (blue, held constant as in neat C_{60} film), the first charge component (orange, Charge 1) and the second charge component (green, Charge 2), expressed as the extinction coefficient of the three species.

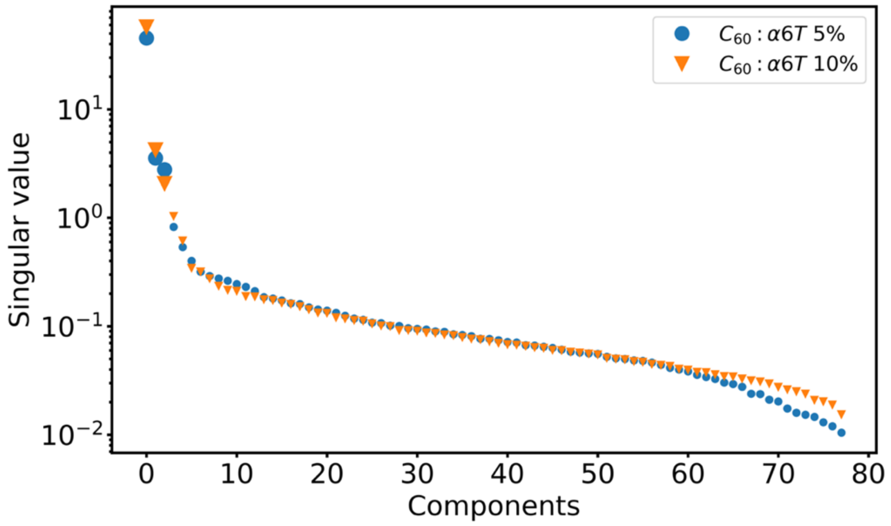

Supplementary Figure 6. SVD analysis of the TA data from the 'dilute-donor' blends. Ordered singular values from the singular value decomposition of the TA spectra of C_{60}:α6T 5% (blue circles) and 10% (orange triangles) blends, measured on the VIS and NIR1 spectrometers. The three most significant singular values are shown with increased marker size.

Comment: The number of components into which the TA spectrum of an OPV system can be decomposed is best determined by an understanding of the experimental conditions and material system, as well as a qualitative analysis of the spectrum. In order to minimize the number of potential components, the C_{60}:α6T blends were excited in a way that only Frenkel type C_{60} excitons are present. After hole transfer, at the interface, the exciton is split into bound and free charges which normally have indistinguishable spectra. The expected number of components is therefore two: one exciton and one charge. However, after performing SVD[3] on the TA data matrices, it is observed that the singular value of the second and third components (shown in **Supplementary Figure 6**) are both significant suggesting that there may be more than two components contributing to the TA spectra.[4]

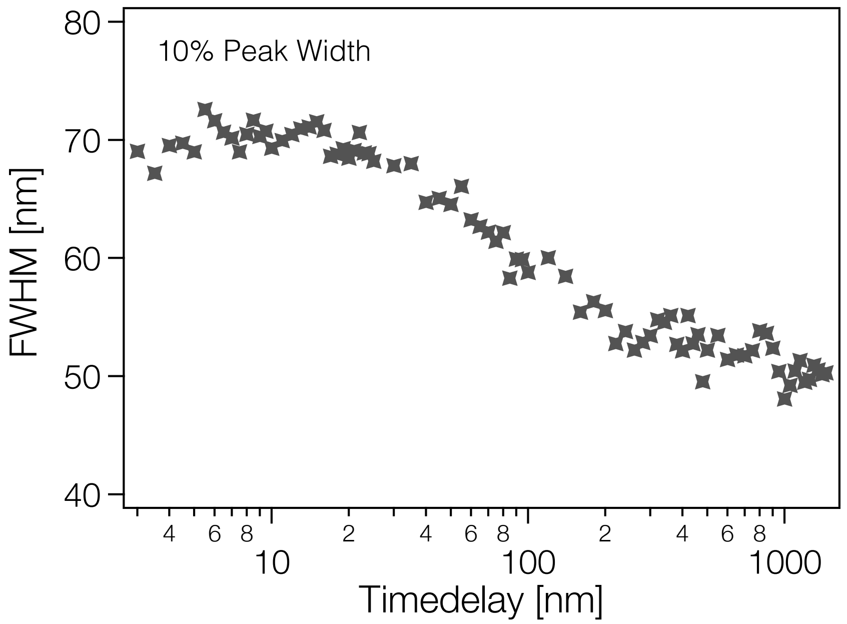

Supplementary Figure 7. Width of the α6T cation band. Temporal evolution of the width (FWHM) of α6T cation band in the TA spectra of the 10% C_{60}:α6T blend excited with $3.7x10^{19}$ cm^{-3} at 610 nm (NIR2).

Comment: The α6T cation band in the TA spectra at all time delays was fit with a Gaussian function for the 5% and 10% blends and bilayer, at the highest available fluence in the NIR2 range. The peak position is shown in the main text (Figure 2B). In **Supplementary Figure 7**, the temporal evolution of the band width (FWHM) of the 10% blend is shown, indicating a spectral narrowing. The width determination for the 5% and bilayer were challenging due to the lower excitation density and stronger mixing with the C_{60} exciton signal.

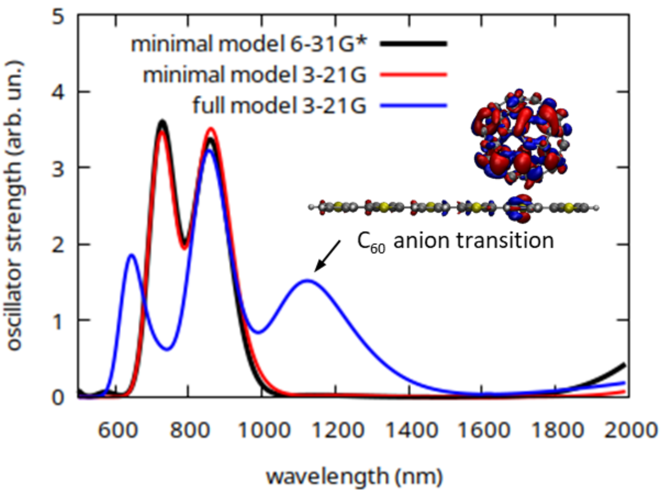

Supplementary Figure 8. Point charge model versus explicit C_{60} anion. Comparison of charge-transfer-state absorption (full model, blue) and cationic α6T with negative point charge (minimal model) for the face-on configuration (see inset and Figure 3C of main manuscript).

Comment: To test our "minimal model" in which the C_{60} anion is represented as negative point charge, we also considered a "full model" where the C_{60} anion is included explicitly. For the latter case, the charge transfer state is obtained by localizing the charge and spin density of one excess electron or hole on the C_{60} and α6T subunits accordingly by means of constrained DFT (cDFT). For this situation, we then performed TD-DFT calculations. The result in **Supplementary Figure 8** shows that both the full model and our minimal model give the same qualitative result regarding the interpretation we give in the main manuscript. That is the broad bimodal spectrum is also present in the full model and therefore confirms our reasoning.

The simulations at the cDFT+TD-DFT level are numerically extremely challenging and cannot be repeated for the large number of different C_{60} positions which we present in the manuscript (Figure 3) for the minimal model. For comparison, the minimal model requires the calculation of the first 20 transition to ensure convergence in the energy region of interest whereas the full model requires 100 transitions or more. On top of that, increasing the number of basis functions (for the larger number of atoms) leads to another large multiplication factor for the computing resources. To be able to carry out the "full model" simulation, we use a smaller basis set (3-21G instead of 6-31G*). As one can see from Figure S8, this results in a nearly identical spectrum for the minimal model (red curve vs black curve), except the fact that the empirical red shift that we apply has to be changed by 0.08 eV. The total computational demands for the full-model simulation with 3-21G basis were >30 times computationally more expensive for a single position of C_{60}. Note that the spectra in Figure 3B-D of the main manuscript were obtained by 100 different orientations each and cannot be repeated in the full model.

There are two major differences between the minimal and the full model: First, an additional peak is formed at around 1100 nm in the full-model simulation, and second, the peak at 730 nm appears about 50 nm blue shifted and of lower intensity for the full model. The former can be attributed to a C_{60} anion absorption. The inset of Figure S9 shows the transition density of the additional peak which is clearly localized at the C_{60} anion (polaron absorption of the C_{60} anion). The second difference, on the other hand, can be rationalized by the electrostatic interaction between the densities constrained at α6T and C_{60} due to which the center of negative charge at the C_{60} is pulled towards the α6T cation resulting in a stronger symmetry break than in the point-charge model. Note that a stronger blueshift at simultaneous lower intensity would enhance the agreement between theory and experiment (see Figure 4A in the main manuscript).

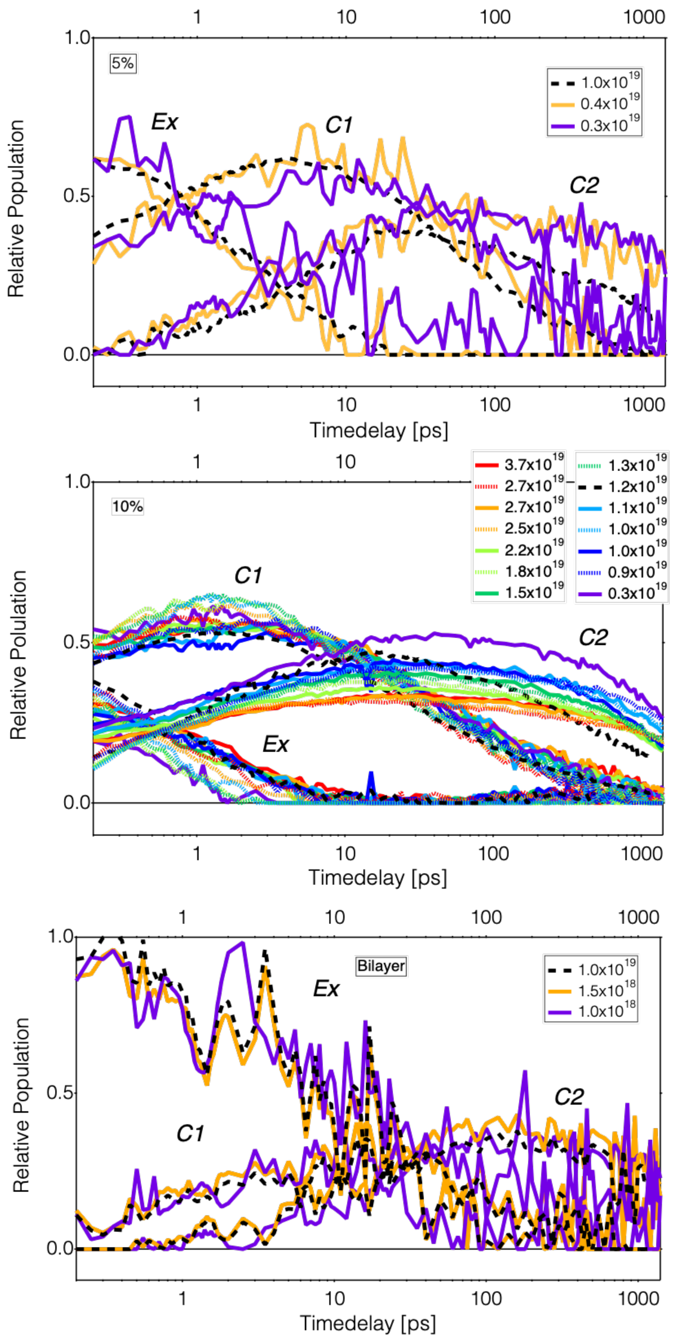

Supplementary Figure 9. Transient absorption dynamics from different measurement series. Dynamics of the three components of the MCR-ALS decomposition in each of the blends and bilayer for every recorded measurement, normalized by the excitation density (shown in the legend in cm^{-3}) to give the relative population. Top: 5% C_{60}:α6T blend recorded with VIS/NIR1 (dashed black line) and NIR2 (solid lines). Middle: 10% C_{60}:α6T blend recorded with VIS/NIR1 (dashed black line) and two NIR2 measurement series (solid and dotted lines). Bottom: C_{60}:α6T bilayer recorded with VIS/NIR1 (dashed black line) and NIR2 (solid lines). Three different spectrometers were used to cover different spectral regions (VIS: 470-720nm, NIR1: 840-1150nm, NIR2: 690-1600 nm), and data was recorded for different excitation densities. Due to experimental constraints, it was more difficult to reach high densities with the NIR2 setup. A high consistency is seen between different measurements, and clear trends with excitation density appear within each measurement series

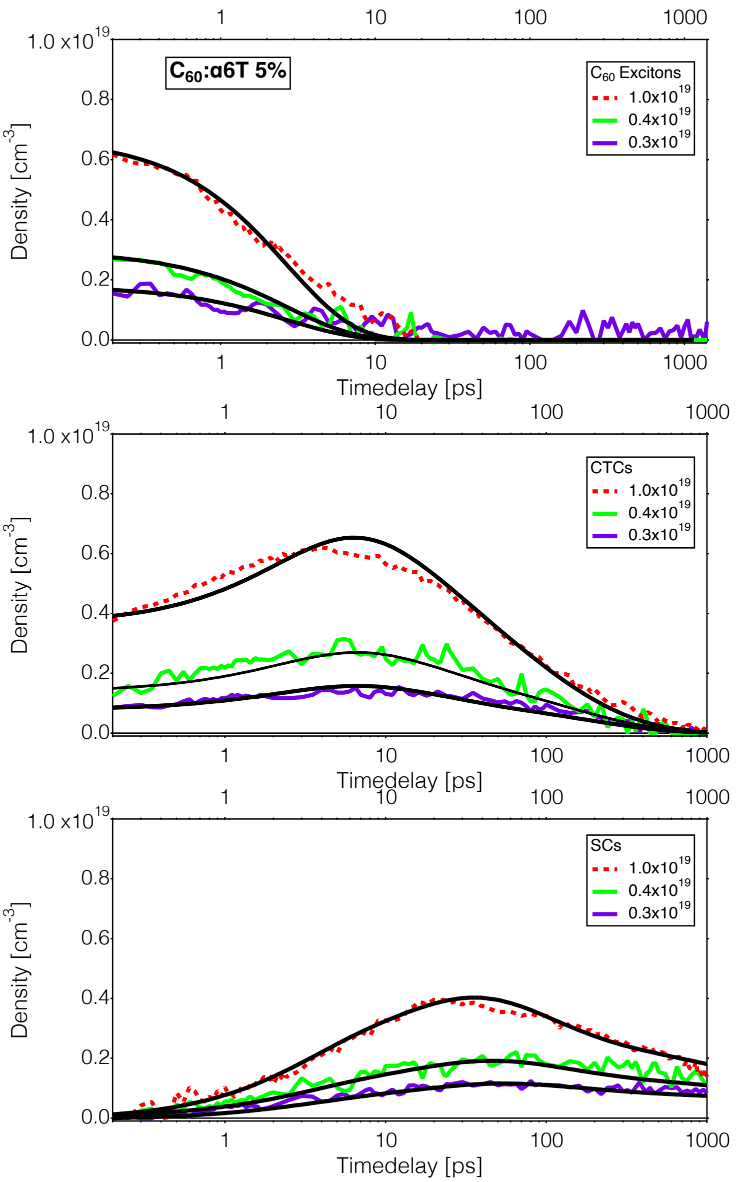

Supplementary Figure 10. Kinetic modelling for the 5% blend. Dynamics of the S_1 exciton (top), CTCs (middle) and SCs (bottom) populations for the 5 % α6T:C_{60} blend as determined by MCR-ALS decomposition, together with the fits (black lines) obtained from the kinetic modelling at various excitation densities (shown in the legend in cm^{-3}). Dashed line: recorded in VIS/NIR1 range, solid lines: recorded with the NIR2 spectrometer.

Comment: The kinetic modelling, as described in the main text, was carried out globally for all excitation densities and for different measurement series recorded with different spectrographs, adding robustness to the results. The fits to the MCR species dynamics are shown in **Supplementary Figures 10-12** and reproduce the experimental data very well. Most excitation densities were recorded for the 10% blend. Here, we note a less good fit at the highest densities, especially for the SCs. The reason is the increased experimental uncertainty at the higher densities, as the pump beam had to be strongly focussed, leading to slight beam walk and changes in the beam profile with timedelay. Therefore, densities >$1.5x10^{19}$ cm^3 were not included in the global analysis of the 10% blend but simulated with the parameters obtained at lower fluences.

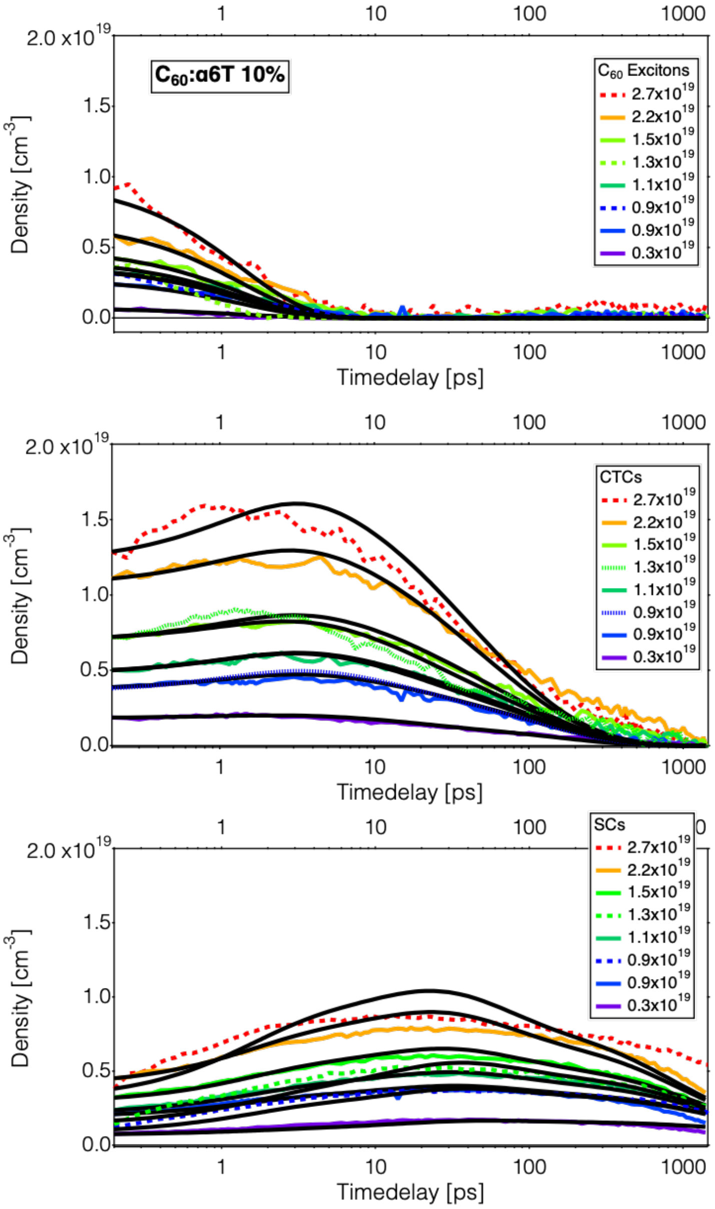

Supplementary Figure 11. Kinetic modelling for the 10% blend. Dynamics of the S_1 exciton (top), CTCs (middle) and SCs (bottom) populations for the 10 % α6T:C_{60} blend as determined by MCR-ALS decomposition, together with the fits (black lines) obtained from the kinetic modelling at various excitation densities (shown in the legend in cm^{-3}). Dashed lines and solid lines are two measurement series recorded with the NIR2 spectrometer.

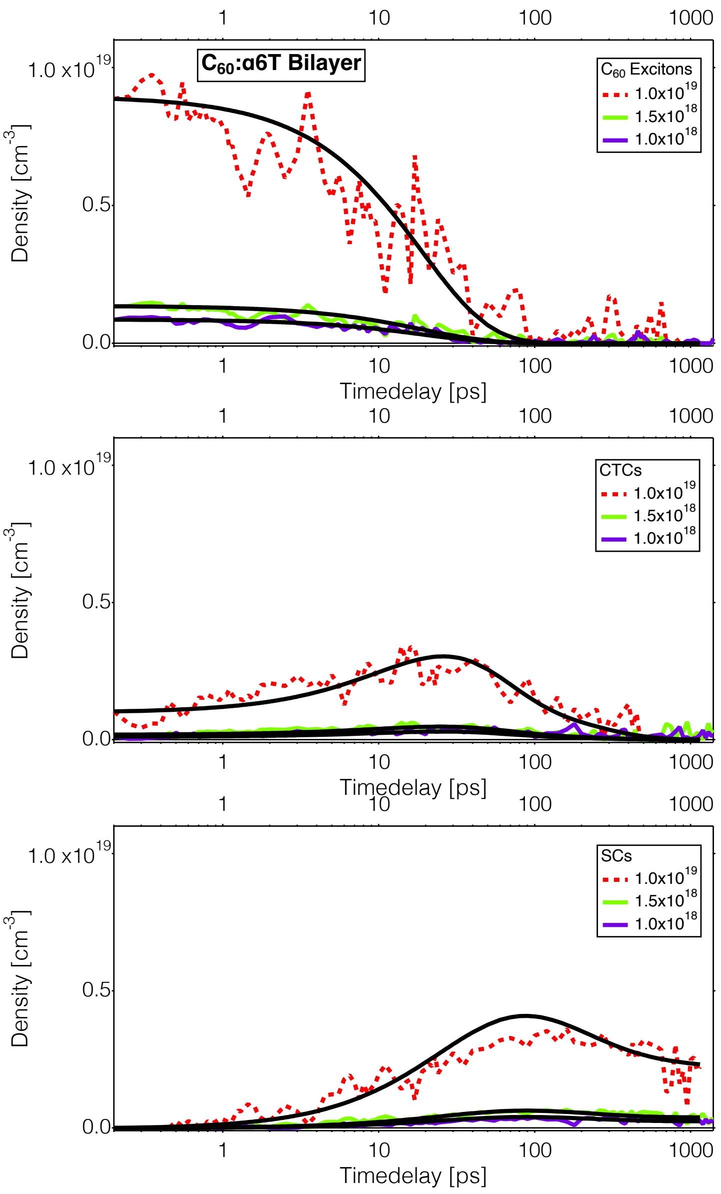

Supplementary Figure 12. Kinetic modelling for the bilayer sample. Dynamics of the S_1 exciton (top), CTCs (middle) and SCs (bottom) populations for the α6T:C_{60} bilayer as determined by MCR-ALS decomposition, together with the fits (black lines) obtained from the kinetic modelling at various excitation densities (shown in the legend in cm^{-3}). Dashed line: recorded in VIS/NIR1 range, solid lines: recorded with the NIR2 spectrometer.

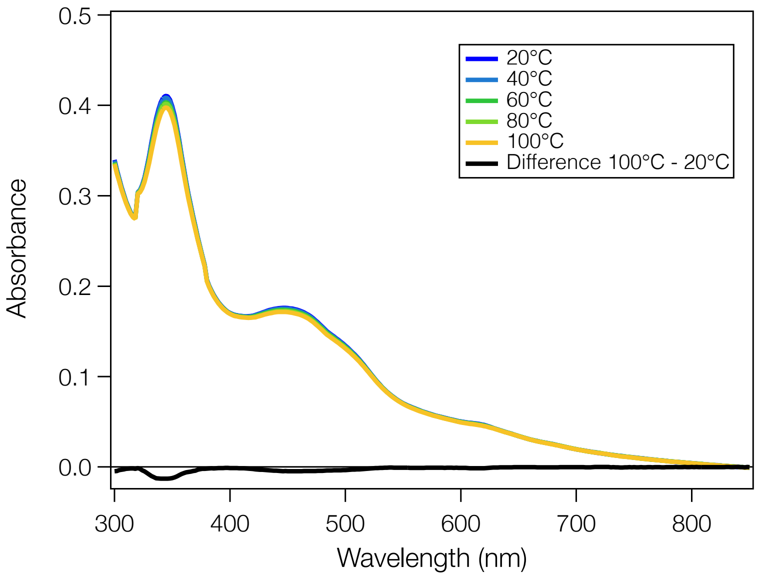

Supplementary Figure 13. Temperature-dependent absorption spectra. Steady-state absorbance spectra of the α6T:C_{60} systems (10%) at different temperatures. This was recorded to mimic any heating effects occurring during the TA spectroscopy. The differential spectrum between 100°C and 20°C represents possible thermal effects occurring between pumped and non-pumped shots in the TA. The effects are weak and occur below 500 nm (outside our TA measurement window).

Supplementary References

1. Causa' M, Ramirez I, Martinez Hardigree JF, Riede M, Banerji N. Femtosecond Dynamics of Photoexcited C60 Films. *The Journal of Physical Chemistry Letters* **9**, 1885-1892 (2018).

2. Moore GJ, *et al*. Ultrafast Charge Dynamics in Dilute-Donor versus Highly Intermixed TAPC:C60 Organic Solar Cell Blends. *The Journal of Physical Chemistry Letters* **11**, 5610-5617 (2020).

3. Van Loan CF. Generalizing the Singular Value Decomposition. *SIAM Journal on Numerical Analysis* **13**, 76-83 (1976).

4. Benke S, Nettels D, Hofmann H, Schuler B. Quantifying kinetics from time series of single-molecule Förster resonance energy transfer efficiency histograms. *Nanotechnology* **28**, 114002 (2017).
